# Phthalate Concentrations and Dietary Exposure from Food Purchased in New York State

**DOI:** 10.1289/ehp.1206367

**Published:** 2013-03-06

**Authors:** Arnold Schecter, Matthew Lorber, Ying Guo, Qian Wu, Se Hun Yun, Kurunthachalam Kannan, Madeline Hommel, Nadia Imran, Linda S. Hynan, Dunlei Cheng, Justin A. Colacino, Linda S. Birnbaum

**Affiliations:** 1 University of Texas School of Public Health, Dallas, Texas, USA; 2 National Center for Environmental Assessment, Office of Research and Development, U.S. Environmental Protection Agency, Washington, DC, USA; 3 Wadsworth Center, New York State Department of Health, Albany, New York, USA; 4 Department of Environmental Health Sciences, State University of New York at Albany, Albany, New York, USA; 5 University of Texas Southwestern Medical Center, Dallas, Texas, USA; 6 University of Michigan, Ann Arbor, Michigan, USA; 7 National Cancer Institute, and; 8 National Institute of Environmental Health Sciences, National Institutes of Health, Department of Health and Human Services, Research Triangle Park, North Carolina, USA

**Keywords:** BBzP, DEHP, DEP, DiBP, market basket survey, phthalate exposure

## Abstract

Background: Phthalates have been found in many personal care and industrial products, but have not previously been reported in food purchased in the United States. Phthalates are ubiquitous synthetic compounds and therefore difficult to measure in foods containing trace levels. Phthalates have been associated with endocrine disruption and developmental alteration.

Objectives: Our goals were to report concentrations of phthalates in U.S. food for the first time, specifically, nine phthalates in 72 individual food samples purchased in Albany, New York, and to compare these findings with other countries and estimate dietary phthalate intake.

Methods: A convenience sample of commonly consumed foods was purchased from New York supermarkets. Methods were developed to analyze these foods using gas chromatography–mass spectroscopy. Dietary intakes of phthalates were estimated as the product of the food consumption rate and concentration of phthalates in that food.

Results: The range of detection frequency of individual phthalates varied from 6% for dicyclohexyl phthalate (DCHP) to 74% for di-2-ethylhexyl phthalate (DEHP). DEHP concentrations were the highest of the phthalates measured in all foods except beef [where di-*n*-octyl phthalate (DnOP) was the highest phthalate found], with pork having the highest estimated mean concentration of any food group (mean 300 ng/g; maximum, 1,158 ng/g). Estimated mean adult intakes ranged from 0.004 μg/kg/day for dimethyl phthalate (DMP) to 0.673 μg/kg/day for DEHP.

Conclusions: Phthalates are widely present in U.S. foods. While estimated intakes for individual phthalates in this study were more than an order of magnitude lower than U.S. Environmental Protection Agency reference doses, cumulative exposure to phthalates is of concern and a more representative survey of U.S. foods is indicated.

Phthalates are diesters of phthalic acids, a class of industrial chemicals extensively used since the early 20th century as softeners of plastics, solvents in perfumes, and additives to hairsprays and lubricants and as insect repellents. Di-2-ethylhexyl phthalate (DEHP) is used primarily as a plasticizer for polyvinyl chloride (PVC) and can therefore be found in a variety of products such as floor and wall coverings, vinyl gloves, toys, child care articles, food packaging materials, and medical devices (Green et al. 2005). After absorption, the parent diester phthalates are rapidly hydrolyzed to the corresponding monoesters, some of which are then further metabolized, with the metabolites excreted in urine and feces. In humans, phthalates are eliminated mostly within hours, with excretion complete by a day or two; half-lives in the body are in hours (Koch and Calafat 2009). For phthalates with short alkyl chains, monoesters represent the major human metabolite, but in the case of phthalates with long alkyl chains, including DEHP, diisononyl phthalate (DINP) and diisodecyl phthalate (DIDP), the monoesters are further metabolized via ω- and ω-1-oxidation of the aliphatic side chain (Agency for Toxic Substances and Disease Registry 2002).

In animal studies, phthalates have been associated with a variety of deleterious health effects. Certain phthalates are reproductive toxicants, especially affecting the male reproductive system (Foster et al. 2000). In rats, phthalate exposure resulted in decreased testicular weight and seminiferous tubular atrophy (Kamrin 2009). Among adult men, urinary phthalate metabolite concentration has been associated with increased DNA damage in sperm (Duty et al. 2003). A possible association between premature breast development and early phthalate exposure in girls has been reported, based on a study comparing serum phthalate concentrations from 41 cases with 35 control samples (Colón et al. 2000). In women, shortened pregnancy has been associated with phthalate exposure; average gestational age at birth was significantly shorter in 65 newborns with detectable mono-2-ethylhexyl phthalate (MEHP) in cord serum compared with 19 newborns with MEHP-negative cord serum (Latini et al. 2003). Prenatal phthalate exposure is associated with a decrease in anogenital distance among male infants (Swan et al. 2005).

Existing exposure pathway assessments for phthalates have included consideration of food and water ingestion, soil and dust ingestion, dermal contact (personal care products, toys, textiles, gloves, paints/adhesives, and dust particles), and inhalation (indoor/outdoor air, hair/paint sprays). Phthalates can migrate into food through the production, packaging, and preparation of food. In a review of the literature, Cao and Xu-Liang (2010) identified sources of phthalates in food including PVC tubing used in food production, food-packaging films (also known as cling films), PVC gaskets in jars, printer inks on labels, and other sources. A recent and comprehensive exposure pathway assessment was conducted by Clark et al. (2011) using data from studies conducted in several countries. Food exposures were estimated primarily based on duplicate diet composite studies in which phthalates were measured in samples of all food and drink consumed by participants. For example, Fromme et al. (2007) collected duplicate diet samples for 50 individual participants 14–60 years of age over 7 days and measured them for several phthalates. Other duplicate diet studies include an analysis of 63 1-week hospital diet samples by Tsumura et al. (2001) and a study of 29 total diet samples and 11 baby food or infant formula samples by Petersen and Breindahl (2000). Duplicate diet studies provide the best data for overall dietary exposure assessment because they capture exposures from food as prepared and eaten. However, these studies are costly and usually test only a small number of individuals and thus may not be representative of the larger population in that country. A weakness of total diet studies is that they do not indicate the type of food products that are major contributors to contaminant exposures. On the other hand, analysis of individual food items for phthalates can provide such information. Total diet studies and also market basket surveys of phthalates in food, such as the one reported here, are rare. Some surveys have been conducted in Europe, Canada, and China [e.g., see Fierens et al. 2012; Food Standards Agency (FSA) 2012; Guo et al. 2012; Page and Lacroix 1995; Wormuth et al. 2006]; results of these studies are compared with the results of the present analysis.

Phthalate measurement in food is difficult because of the ubiquitous occurrence of phthalates in many products, including analytical solvents as well as laboratory air and equipment. Although some phthalate food concentration studies appear in the scientific literature, the results may suffer from the issue of ubiquitous contamination. To our knowledge, phthalate concentrations in foods from the United States have not previously been reported. The purpose of this study was to describe for the first time the presence and concentrations of phthalates in foods as purchased at U.S. supermarkets. In this study, we collected a convenience sample of 72 commonly consumed foods from supermarkets in Albany, New York, for the analysis of nine phthalate esters. Additional care was taken to insure that potential laboratory contamination would not influence the results, including the subtraction of blank concentrations from sample measurements [see Guo and Kannan (2012) for a review of contamination issues and how we resolved those concerns]. These issues are further addressed later in this report.

## Methods

*Sample collection*. A convenience sample of 72 commonly consumed food samples [see Supplemental Material, Table S1 (http://dx.doi.org/10.1289/ehp.1206367)] was purchased from supermarkets in Albany, New York, in April 2011. Samples were frozen at –20°C after purchase. Among the 72 foods that were measured, 65 were grouped into ≥ 1 food categories (Table 1) and 7 were not included in any of the 13 food categories under consideration because they were mixtures and difficult to place in food categories (e.g., chicken pot pie, pizza, onion soup mix).

**Table 1:** Food group consumption rate and group sample content.

Food group	Consumption rate (g/kg/day)a	No. of samplesb	Group sample content	Container type
Beveragesc	13.0	8	Diet lemon tea, lemonade, soda, bottled water, green tea, apple juice, sports drink (80 calories) and sports drink (0 calories)	All in plastic
Milk	2.3	2	Two brands of milk	All in plastic
Other dairy	1.2	9	Pudding, sliced cheese, shredded cheese and two brands each of ice cream, butter, and yogurt	Two ice creams and two butters in paper; the rest in plastic
Fish	0.23	5	Salmon fillet, tuna, raw shrimp, chopped clams, and sardines	Salmon in foam tray and plastic wrap; tuna and shrimp in paper; the rest in metal
Fruits/vegetables	3.4	5	Two brands of vegetable soups, two brands of canned fruits, and canned tomato sauce	All in plastic except for tomato sauce and canned fruits in metal
Grain	2.2	7	Bread, cake mix, cereals, rice, three brands of cookies	Bread and one brand of cookies in plastic; the rest in paper
Beef	0.73	2	Ground beef and beef	Ground beef in plastic and paper; beef in foam tray with a pad and plastic wrap
Pork	0.36	4	Sausage links, pork, pork bacon, and ham	Pork bacon in plastic and paper; ham in plastic; the rest in foam tray and plastic wrap
Poultry	0.71	6	Ground turkey, ground chicken, chicken drumsticks, turkey breast, turkey bacon, and chicken franks	Turkey bacon in plastic and paper; turkey breast and chicken franks in plastic; the rest in foam tray and plastic wrap
Meat and meat productsd		13	Hot dog plus all 12 food samples in above beef, pork, and poultry groups	Hot dog in plastic; other container types as listed in above beef, pork, and poultry
Vegetable oils	0.39	3	Canola oil, virgin olive oil, and vegetable oil	Virgin olive oil in glass; the rest in plastic
Condiments	0.2e	6	Pancake syrup, marinade, barbecue sauce, Italian dressing, ranch dressing, and tomato ketchup	Marinade in glass; the rest in plastic
Infant foodsf	56.0	7	Rice cereal, vegetable homogenate, food homogenate, two brands of fruit homogenate, ready-made meal for babies (chicken/carrot ravioli), and ready-made meal for babies (cheese ravioli)	Rice cereal in paper; food homogenate and fruit homogenate in glass; the rest in plastic
aAll consumption rates are from the Exposure Factors Handbook (U.S. EPA 2011b), except condiments, which are from Dinovi and Brookmire (2011), and infant foods, from U.S. EPA (2008). bTotal n = 65, 12 of 13 samples included in “meat and meat products” group are included in other groups as well. Also, 7 other samples (for a total n of 72 in the survey) were not included in any food group; see text for more detail. cAssumes 1.4 L/day average adult water consumption for 70 kg adult. dIncludes all samples included in the beef, pork, and poultry groups plus 1 hot dog sample (not included in any other group); concentration assumed to be the average of all meat concentrations. eBased on 11.5 g/day provided by Dinovi and Brookmire (2011) divided by an assumed adult body weight of 70 kg. fTotal infant foods not including breast milk, ages 6 months to 1 year from the U.S. EPA (2008).				

*Analytical methods*. Each sample was individually analyzed for nine phthalates: dimethyl phthalate (DMP), diethyl phthalate (DEP), butyl benzyl phthalate (BBzP), di-*n*-butyl phthalate (DBP), diisobutyl phthalate (DiBP), DEHP, di-*n*-hexyl phthalate (DnHP), dicyclohexyl phthalate (DCHP), and di-*n*-octyl phthalate (DnOP). All glassware was baked at 450°C overnight. A detailed report on the analysis and quality assurance and quality control methods is given elsewhere (Guo and Kannan 2012; Guo et al. 2012). All food samples (1.5–20 g), except soft drinks, were freeze-dried using a freeze drier (Labconco, Kansas City, MO), ground to a fine powder using a precleaned mortar and pestle, and spiked with 50 ng deuterated internal standards [D_4_-DMP, D_4_-DEP, D_4_-DnHP (for BBzP), D_4_-DCHP, D_4_-DBP, D_4_-DiBP, D_4_-DEHP and D_4_-DnOP] (AccuStandard Inc., New Haven, CT). For liquid samples that did not contain lipids, 50 g samples were extracted three times with 15 mL hexane by shaking for 30 min. The combined extracts were concentrated to 2 mL using a rotary evaporator. For solid foods, the ground samples were extracted with 20 mL acetone:hexane (1:1, vol:vol) three times, and centrifuged after shaking for 30 min. The upper organic layers were combined, concentrated to 1 mL, and transferred to 35 mL glass tubes with PTFE (Teflon) caps. After adding 30 mL hexane–saturated acetonitrile solution and 3 mL hexane, samples were shaken for 10 min and the upper layer was discarded (this was repeated three times). For cookies, which contained large amounts of lipids, after three extractions, the extract was placed in a refrigerator for half an hour and the upper layer was transferred into another tube for further concentration. The extracts were concentrated to near dryness, and then 2 mL hexane was added for further clean up by column chromatography.

A glass column packed with 7 g Florisil 60–100 mesh (Aldrich, St. Louis, MO) was used for the purification of extracts. Phthalates were eluted with 70 mL acetone:hexane (2:8, vol:vol). The final eluate was concentrated to 0.5 mL under a gentle stream of nitrogen for gas chromatography–mass spectrometry (GC-MS) analysis. For each batch of 10 samples, 3 method blanks—a spiked blank and a pair of matrix-spiked samples (spiked with 100 ng each of target phthalates) per duplicate—were processed. When the concentrations of DEHP and DBP in the 3 procedural blanks varied widely, and if the difference in concentrations among the blanks exceeded 50 ng, then all the data were discarded, and samples were reanalyzed. Mean blank values were subtracted from sample values for each batch.

*Instrumental analysis*. The nine phthalates were measured using a gas chromatograph (Agilent Technologies 6890N; Agilent Technologies, Santa Clara, CA) coupled with a mass spectrometer (Agilent Technologies 5973) (GC-MS) in the selective ion monitoring mode. A fused-silica capillary column (DB-5; 30 m × 0.25 mm i.d.; 0.25 mm film thickness) was used for separation. Samples (1 µL) were injected in the splitless mode. The temperatures of the injector and ion source were 280°C and 230°C, respectively. The oven temperature was programmed from 80°C (held for 1.0 min), raised to 180°C at 12°C/min (held for 1.0 min), increased to 230°C at 6°C/min, then to 270°C at 8°C/min (held for 2.0 min), and finally to 300°C at 30°C/min (held for 12 min). Ion fragments *m/z* 163, *m/z* 279, and *m/z* 149 were monitored for DMP, DnOP, and the other seven phthalates, and fragment *m/z* 206 was used for confirmation of BBzP. Deuterated internal standards for each phthalate were used for quantification. DiBP, DBP, and DEHP were present in all procedural blanks (*n* = 17) at mean concentrations of 0.2 (range, 0.04–0.31), 1.4 (range, 0.16–8.0), and 3.7 (range, 0.53–7.4) ng/g, respectively. These values were subtracted from sample values. The limits of detection (LODs) were 1.4, 3.7, and 1.0 ng/kg for DBP, DEHP, and DnOP, respectively, and 0.2 ng/kg for the other phthalates.

*Dietary intake*. Dietary intakes for adults were estimated for major groups of foods (e.g., fruits/vegetables, grain, beef) (Table 1), and intakes for infants were estimated based on infant food samples. Two different intakes were derived, one as the product of a mean food group consumption rate and a mean food group concentration, and the second as the product of a mean food group consumption rate and the median food group concentration. Food group concentrations were derived using one-half the LOD as the value for samples below the LOD, and after substituting 0 for values < LOD. Dietary intake estimates were very similar for both approaches; therefore, we report intake estimates using one-half LOD only. The food consumption rates for adults were those recommended for estimating general adult population exposures by the 2011 *Exposure Factors Handbook* (EFH) based on the U.S. Environmental Protection Agency (EPA) analyses of National Health and Nutrition Examination Survey (NHANES) 2003–2006 data (U.S. EPA 2011b). The “condiments” food group included pancake syrup, marinade, barbecue sauce, Italian dressing, ranch dressing, and tomato ketchup samples. A total daily consumption rate of 11.5 g/day was not provided in the EFH but was instead derived using the U.S. Department of Agriculture (USDA) Food and Nutrient Database for Dietary Studies (FNDDS 4.1; Bodner-Montville et al. 2006), which is the database associated with NHANES (Dinovi and Brookmire 2011). The food consumption rate for infants was the mean consumption rate for infants between 6 and 12 months of age from the U.S. EPA’s *Child-Specific Exposure Factors Handbook* (U.S. EPA 2008).

## Results

Consumption rates of each food group, in units of grams per kilogram per day, are shown in Table 1, along with the individual food products constituting each food group and the container types for the individual food samples. Of the food groups evaluated for adults, condiments had the lowest estimated consumption rate (0.2 g/kg/day) and beverages had the highest (13 g/kg/day). The estimated consumption rate for infant foods was 56 g/kg/day.

The frequency of detection of phthalates in various food types varied from zero to 100% (Table 2). Despite this, we detected at least some phthalates in every food group in this study. DEHP was detected in 74% of food samples, including all seven infant food samples, whereas DCHP was detected in only 6% of food samples (Table 2). DEHP was not detected in the two beef samples, but was found in three of four samples of pork, five of six samples of chicken, and in the hot dog sample, which was not included in any other food group. Other phthalate esters were detected at the following frequencies: DEP (57%), DiBP (55%), BBzP (54%), DMP (37%), and DBP (31%).

**Table 2:** Detection frequencies [*n* (%)] of phthalate esters by food group from Albany, New York.

Food	No. of samples	DMP	DEP	DiBP	DBP	DnHP	BBzP	DCHP	DEHP	DnOP
Beverages	8	2 (25)	0	3 (38)	0	0	0	0	1 (13)	0
Milk	2	0	1 (50)	1 (50)	1 (50)	0	1 (50)	0	2 (100)	1 (50)
Other dairy	9	4 (44)	6 (67)	7 (78)	5 (56)	3 (33)	6 (67)	1 (11)	9 (100)	1 (11)
Fish	5	2 (40)	3 (60)	2 (40)	2 (40)	1 (20)	2 (40)	0	4 (80)	0
Fruits/vegetables	5	0	1 (20)	4 (80)	0	0	2 (40)	0	2 (40)	0
Grain	7	5 (71)	7 (100)	6 (86)	6 (86)	3 (43)	7 (100)	0	7 (100)	0
Beef	2	1 (50)	2 (100)	0	0	1 (50)	1 (50)	0	0	1 (50)
Pork	4	2 (50)	4 (100)	1 (25)	0	0	1 (25)	0	3 (75)	1 (25)
Poultry	6	3 (50)	5 (83)	0	0	1 (17)	2 (33)	0	5 (83)	0
Meat and meat products	13	7 (54)	12 (92)	1 (8)	0	2 (15)	4 (31)	0	9 (69)	2 (15)
Vegetable oils	3	1 (33)	0	2 (67)	1 (33)	1 (33)	3 (100)	1 (33)	2 (67)	1 (33)
Condiments	6	3 (50)	3 (50)	5 (83)	3 (50)	0	4 (67)	1 (17)	5 (83)	1 (17)
Infant food	7	0	4 (57)	5 (71)	2 (29)	0	6 (86)	1 (14)	7 (100)	2 (29)
Total	65a	24 (37)	37 (57)	36 (55)	20 (31)	10 (15)	35 (54)	4 (6)	48 (74)	8 (12)
LODs (wet weight): DBP = 1.4, DEHP = 3.7, DnOP = 1.0, all others = 0.2 ng/g. aTotal number of individual samples, does not include samples in more than one group.										

DEHP had the highest concentration of the phthalates tested for all food categories except beef and vegetable oils, ranging from a mean of 4 ng/mL for beverages to 300 ng/g for pork (Table 3). As noted above, DCHP was the least frequently detected phthalate, quantified in only 4 of 65 samples (one in each of four food groups), including 3 samples with concentrations ≤ 1.9 ng/g, and 1 olive oil sample with a concentration of 42.6 ng/g [see Supplemental Material, Table S1 (http://dx.doi.org/10.1289/ehp.1206367)]. Median concentrations were consistently lower than mean concentrations, and for some food groups the discrepancy was large. For example, the mean value of BBzP in vegetable oil (154 ng/g, compared with the median value of 2.2 ng/g) (Table 3) was influenced by a single olive oil sample with a high concentration (459 ng/g) relative to the other oil samples (0.35 and 2.2 ng/g) (see Supplemental Material, Table S1). The olive oil sample was contained in a glass jar, whereas the canola and vegetable oil samples were contained in plastic containers. The concentration of DEHP in the olive oil sample was also high (300 ng/g). DBP was found in 5 of 9 samples of “other dairy” (dairy products other than milk, including 2 cheese samples with concentrations of 138 and 513 ng/g), and in 6 of 7 grain samples (average concentration = 16 ng/g). Other noteworthy individual samples included a ham sample with 1,158 ng/g DEHP and a bread sample with 78.8 ng/g DEP. Overall, these data indicate substantial heterogeneity in phthalate concentrations, even within food groups.

**Table 3:** Mean and median food group concentrations (ng/g whole weight)^*a*^ of phthalate esters from Albany, New York.

Food	Statistic	DMP	DEP	DiBP	DBP	DnHP	BBzP	DCHP	DEHP	DnOP
Beverages	Mean	0.13/0.06	0.1/0	0.29/0.23	0.7/0	0.1/0	0.1/0	0.1/0	3.89/2.28	0.5/0
	Median	0.1/0	0.1/0	0.1/0	0.7/0	0.1/0	0.1/0	0.1/0	1.85/0	0.5/0
Milk	Mean	0.1/0	0.17/0.12	0.2/0.15	1.5/1.15	0.1/0	0.55/0.5	0.1/0	48.6/48.6	1.51/1.26
	Median	0.1/0	0.17/0.12	0.2/0.15	1.5/1.15	0.1/0	0.55/0.5	0.1/0	48.6/48.6	1.51/1.26
Other dairy	Mean	0.48/0.42	1.37/1.34	1.91/1.89	105/104.4	1.25/1.18	4.22/4.19	0.3/0.21	144/144	2.76/2.31
	Median	0.1/0	0.66/0.66	0.79/0.79	4.77/4.77	0.1/0	1.2/1.2	0.1/0	92.8/92.8	0.5/0
Fish	Mean	0.21/0.15	0.6/0.56	1/0.94	11/10.6	0.13/0.05	1.61/1.55	0.1/0	31.7/31.4	0.5/0
	Median	0.1/0	0.86/0.86	0.1/0	0.7/0	0.1/0	0.1/0	0.1/0	39.6/39.6	0.5/0
Fruit/vegetables	Mean	0.1/0	0.12/0.04	0.55/0.53	0.7/0	0.1/0	0.67/0.61	0.1/0	6.2/5.09	0.5/0
	Median	0.1/0	0.1/0	0.48/0.48	0.7/0	0.1/0	0.1/0	0.1/0	1.85/0	0.5/0
Grain	Mean	0.3/0.27	12.6/12.6	3.54/3.52	15.9/15.8	0.23/0.17	5.92/5.92	0.1/0	61.6/61.6	0.5/0
	Median	0.34/0.34	1.17/1.17	1.64/1.64	5.14/5.14	0.1/0	4.65/4.65	0.1/0	50.6/50.6	0.5/0
Beef	Mean	0.18/0.13	0.64/0.64	0.1/0	0.7/0	2.47/2.42	0.61/0.56	0.1/0	1.85/0	3.57/3.32
	Median	0.18/0.13	0.64/0.64	0.1/0	0.7/0	2.47/2.42	0.61/0.56	0.1/0	1.85/0	3.57/3.32
Pork	Mean	0.33/0.28	0.55/0.55	6.25/6.18	0.7/0	0.1/0	0.23/0.15	0.1/0	300/300	2.86/2.49
	Median	0.16/0.11	0.59/0.59	0.1/0	0.7/0	0.1/0	0.1/0	0.1/0	20.6/20.6	0.5/0
Poultry	Mean	0.15/0.1	0.41/0.4	0.1/0	0.7/0	0.21/0.12	0.66/0.6	0.1/0	18.6/18.3	0.5/0
	Median	0.15/0.1	0.33/0.33	0.1/0	0.7/0	0.1/0	0.1/0	0.1/0	14.8/14.8	0.5/0
Meat and meat products	Mean	0.22/0.17	0.49/0.48	1.99/1.9	0.7/0	0.51/0.43	0.48/0.41	0.1/0	101.8/101	1.7/1.28
	Median	0.2/0.2	0.45/0.45	0.1/0	0.7/0	0.1/0	0.1/0	0.1/0	7/7	0.5/0
Vegetable oils	Mean	1.2/1.14	0.1/0	3.2/3.17	3.53/3.07	0.19/0.12	154/154	14.27/14.2	117/116.3	0.84/0.5
	Median	0.1/0	0.1/0	0.25/0.25	0.7/0	0.1/0	2.2/2.2	0.1/0	48.9/48.9	0.5/0
Condiments	Mean	0.33/0.28	0.77/0.72	1/0.98	15.4/15	0.1/0	1.99/1.96	0.13/0.05	30.4/30.1	1.19/0.77
	Median	0.2/0.15	0.16/0.11	0.81/0.81	1.6/1.25	0.1/0	1.33/1.33	0.1/0	20.6/20.6	0.5/0
Infant food	Mean	0.1/0	0.35/0.31	0.77/0.74	1.14/0.64	0.1/0	3.36/3.35	0.18/0.1	75.1/75.1	2.5/2.14
	Median	0.1/0	0.28/0.28	0.22/0.22	0.7/0	0.1/0	2.37/2.37	0.1/0	29.4/29.4	0.5/0
aConcentrations are displayed as the phthalate ester concentration in a food group when substituting one-half the LOD for each nondetect ÷ the phthalate ester concentration in a food group when substituting 0 for each nondetect.										

Table 4 shows a comparison of mean phthalate concentrations in food groups from our survey with concentrations previously reported in the literature. One, published by Wormuth et al. (2006), was an assessment of phthalates in Europe, Asia, and North America. They reported on a dozen studies from different countries around the world to derive food and food group–specific concentrations. These studies were published between 1995 and 2005, with half published before 2000. An earlier study by Health Canada (Page and Lacroix 1995) assessed phthalate occurrence in 260 samples of foods, 98 samples of food composites, and samples of food packaging materials. A third was conducted by the Food Standards Agency (FSA) of the United Kingdom and included analysis of 20 composite samples, 29 samples of food packaging materials, and 261 individual samples, all collected in 2005 and later (FSA 2012). Finally, Fierens et al. (2012) reported on a sampling of 400 food products in Belgium in 2009 and 2010. Concentrations found in the present study appear somewhat comparable to those reported by Wormuth et al. (2006) for fish and for values derived by averaging mean values reported by Wormuth and colleagues for groups of beverages and dairy products; however, Wormuth and colleagues’ compilation describes concentrations that are about an order of magnitude higher for meat products for DEHP and DBP. The earlier study from Health Canada (Page and Lacroix 1995) occurred in the late 1980s, so the relevance here is questionable. However, it is certainly noteworthy that it is the only major study of phthalates in food in North America that we found in the literature. BBzP was noted as being not detected in most samples–it was found only in some grain and a few dairy products. DEHP was found most often and at higher concentrations than reported in more recent studies. The one poultry sample had a high concentration of 2,600 ng/g. The detection limits of the FSA (2012) survey were comparable to detection limits achieved here, ranging from about 5 to 20 ng/g. The FSA (2012) results appear to be the most comparable to our data among all the studies shown in Table 4. DEHP was generally the phthalate with the highest concentrations measured in foods in the FSA (2012) survey, as in the present study, and nearly all DEHP concentrations reported for the FSA survey are within a factor of 5 of our data. Results for DBP also compare very favorably between the FSA data and our data, whereas the compilation of Wormuth et al. (2006) reported higher concentrations of this phthalate ester. A similar comparability is seen in the comparison results for BBzP and DiBP between our study and the U.K. study (FSA 2012), with many showing average concentrations of < 1 ng/g or being nondetectable. Generally, the lowest concentrations in food were measured from samples taken in Belgian markets (Fierens et al. 2012), with median values that were often very low or nondetectable. However, similar to the findings of other studies, DEHP was quantified most frequently and at the highest concentrations of the phthalates tested, with median concentrations of 28 ng/g in dairy products, 86 ng/g in fish, and 44.5 ng/g in various meats. However, because DEHP and DBP are the most common contaminants in laboratory solvents and reagents, care should be exercised in quantifying these two phthalate esters (Guo and Kannan 2012).

**Table 4:** Comparison of phthalate food concentrations reported elsewhere in the literature with food concentrations found in the present study (ng/g wet weight).

Food	Source	DEHP	DBP	BBzP	DiBP
Beverages	This study (mean)	3.9	0.7	0.1	0.3
	Wormuth et al. (2006)a	14	18	0.1	2
	Page and Lacroix (1995)b	ND	—	ND	—
	FSA (2012)c: MK	—	—	—	—
	FSA (2012)c: TDS	ND	ND	ND	ND
	Fierens et al. (2012)d	0.1	0.1	0.1	0.1
All dairy	This study (mean)	126.5	85.9	3.6	1.6
	Wormuth et al. (2006)a	211	22	14	0.4
	Page and Lacroix (1995)b	830	—	260	—
	FSA (2012)c: MK	159	ND	ND	12
	FSA (2012)c: TDS	71	ND	ND	ND
	Fierens et al. (2012)d	27.5	2.0	ND	2.4
Fish	This study (mean)	31.7	11.0	1.6	1.0
	Wormuth et al. (2006)a	13	8	5	1
	Page and Lacroix (1995)b	67	—	ND	—
	FSA (2012)c: MK	59	ND	ND	ND
	FSA (2012)c: TDS	789	9	ND	1
	Fierens et al. (2012)d	86.0	ND	ND	ND
Beef	This study (mean)	1.9	0.7	0.6	0.1
	Wormuth et al. (2006)a	207	75	0	7
	Page and Lacroix (1995)b	50	—	ND	—
	FSA (2012)c: MK	34	0.5	ND	ND
	FSA (2012)c: TDS	90	ND	ND	ND
	Fierens et al. (2012)d	44.5	1.5	ND	2.0
Pork	This study (mean)	300	0.7	0.2	6.3
	Wormuth et al. (2006)a	64	4	0	0
	Page and Lacroix (1995)b	250	—	ND	—
	FSA (2012)c: MK	34	0.5	ND	ND
	FSA (2012)c: TDS	90	ND	ND	ND
Poultry	This study (mean)	18.6	0.7	0.7	0.1
	Wormuth et al. (2006)a	518	100	15	30
	Page and Lacroix (2005)b	2,600	—	ND	—
	FSA (2012)c: MK	34	0.5	ND	ND
	FSA (2012)c: TDS	322	ND	ND	ND
Abbreviations: —, no data provided for phthalate/food pair; MK, market basket; ND, searched for, but not found at provided detection limits; TDS, Total Diet Survey. aFood concentrations are compiled in Table IV of Wormuth et al. (2006) and come from a variety of sources available at that time. To get food group averages, the following tabular entries were averaged: Beverages—juices, tea, coffee, soft drinks, beer, wine, spirits, tap water, bottled water; All dairy—milk/milk beverages, cream, ice cream, yogurt, and cheese; Fish—fish/seafood; Beef—meat/meat products; Pork—sausages; Poultry—poultry. bMeasurements in food were reported for DEHP, BBzP, and DiBP in Page and Lacroix (1995). DBP was not studied in Page and Lacroix (1995). cThe Food Services Agency report (FSA 2012) contained average results for a targeted market basket (MK) survey as well as the Total Diet Survey (TDS). The average results (calculated at ND = 0) for these two surveys are provided here as MK/TDS. The market basket survey presented average results for all meat products, including beef, pork, and poultry products; this is why the same phthalate MK results are shown for beef, pork, and poultry. For “grain,” both UK surveys had “bread products” and “miscellaneous cereal products,” and these two categories were averaged for this table. dResults were taken from Table 5 of Fierens et al. (2012). They are medians of sampled foods. In some cases, the median was ND, so ND was put into the table; see Table 5 in Fierens et al. (2012) for the maximum concentration found and other information. The study sampled 13 “meats” and 9 “meat products,” without delineating between beef, pork, or poultry. The results provided under beef are for the 13 “meat” samples, and there are no results provided for pork or poultry.

Table 5 presents the estimated daily intake by phthalate and food group. The total estimated intake based on mean concentrations (with values < LOD imputed as one-half LOD), was highest for DEHP (0.673 μg/kg/day) followed by DBP (0.184 μg/kg/day), whereas estimated total mean intakes of all other phthalates were ≤ 0.1 μg/kg/day. Estimated intakes of all phthalates were fairly comparable between adults and infants on a body weight basis, with the exception of DEHP, where the estimated intake from infant food (4.2 μg/kg/day) was more than twice that of adults. Estimated total intakes were similar when a value of zero was assigned to samples with measured concentrations of below the LOD. As expected, intakes derived using mean concentrations were higher than intakes derived using median concentrations, sometimes by over a factor of two.

**Table 5:** Adult and child daily dietary intakes of phthalate esters (μg/kg/day) for mean and median food group concentrations in Albany, New York.

Food	Statistic	DMP	DEP	DiBP	DBP	DnHP	BBzP	DCHP	DEHP	DnOP
Beverages	Mean	0.002	0.001	0.004	0.009	0.001	0.001	0.001	0.051	0.007
	Median	0.001	0.001	0.001	0.009	0.001	0.001	0.001	0.024	0.007
Milk	Mean	< 0.001a	< 0.001	< 0.001	0.003	< 0.001	0.001	< 0.001	0.112	0.003
	Median	< 0.001	< 0.001	< 0.001	0.003	< 0.001	0.001	< 0.001	0.112	0.003
Other dairy	Mean	0.001	0.002	0.002	0.126	0.001	0.005	< 0.001	0.173	0.003
	Median	< 0.001	0.001	0.001	0.006	< 0.001	0.001	< 0.001	0.111	0.001
Fish	Mean	< 0.001	< 0.001	< 0.001	0.003	< 0.001	< 0.001	< 0.001	0.007	< 0.001
	Median	< 0.001	< 0.001	< 0.001	< 0.001	< 0.001	< 0.001	< 0.001	0.009	< 0.001
Fruits/vegetables	Mean	< 0.001	< 0.001	0.002	0.002	< 0.001	0.002	< 0.001	0.021	0.002
	Median	< 0.001	< 0.001	0.002	0.002	< 0.001	< 0.001	< 0.001	0.006	0.002
Grain	Mean	0.001	0.028	0.008	0.035	0.001	0.013	< 0.001	0.136	0.001
	Median	0.001	0.003	0.004	0.011	< 0.001	0.010	< 0.001	0.111	0.001
Beef	Mean	< 0.001	< 0.001	< 0.001	0.001	0.002	< 0.001	< 0.001	0.001	0.003
	Median	< 0.001	< 0.001	< 0.001	0.001	0.002	< 0.001	< 0.001	0.001	0.003
Pork	Mean	< 0.001	< 0.001	0.002	< 0.001	< 0.001	< 0.001	< 0.001	0.108	0.001
	Median	< 0.001	< 0.001	< 0.001	< 0.001	< 0.001	< 0.001	< 0.001	0.007	< 0.001
Poultry	Mean	< 0.001	< 0.001	< 0.001	< 0.001	< 0.001	< 0.001	< 0.001	0.013	< 0.001
	Median	< 0.001	< 0.001	< 0.001	< 0.001	< 0.001	< 0.001	< 0.001	0.011	< 0.001
Vegetable oils	Mean	< 0.001	< 0.001	0.001	0.001	< 0.001	0.060	0.006	0.046	< 0.001
	Median	< 0.001	< 0.001	< 0.001	< 0.001	< 0.001	0.001	< 0.001	0.019	< 0.001
Condiments	Mean	< 0.001	< 0.001	< 0.001	0.003	< 0.001	< 0.001	< 0.001	0.006	< 0.001
	Median	< 0.001	< 0.001	< 0.001	< 0.001	< 0.001	< 0.001	< 0.001	0.004	< 0.001
TOTAL—adult 1/2 LOD for nondetects	Mean	0.004	0.033	0.020	0.184	0.006	0.085	0.008	0.673	0.021
	Median	0.003	0.007	0.008	0.034	0.004	0.016	0.002	0.416	0.017
Baby food	Mean	0.006	0.020	0.043	0.064	0.006	0.188	0.010	4.203	0.140
	Median	0.006	0.016	0.012	0.039	0.006	0.133	0.006	1.646	0.028
For concentrations < LOD, we assumed a value of one-half LOD. a Phthalate was not detected in ≥ 1 samples in this group, and the intake would be < 0.001 μg/kg/day if substituting one-half LOD for each nondetect.										

## Discussion

Others have estimated phthalate intakes based on measured concentrations in media and consumption rates, consistent with our approach. Most have reported higher estimated intakes, in part because they included nondietary intakes, but also because concentrations measured in food surveys were often higher than measured in our food samples. Wormuth et al. (2006) estimated total phthalate intakes for Europeans using a multi-pathway approach that accounted for food and non-food pathways of exposure, including ingestion of dust, soil, and personal care products; mouthing plastic toys and other products; dermal exposure to personal care products, gloves, and other sources; and inhalation (e.g., of phthalates in indoor and outdoor air and paints). Estimated total intakes from all sources reported by Wormuth and colleagues for DEHP, DBP, BBzP, and DiBP were approximately 3.0, 4.0, 0.4, and 0.6 μg/kg/day, respectively (based on data reported in Figure 5 of Wormuth et al. 2006), compared to estimated total dietary intakes of 0.7, 0.2, 0.1, and 0.02 μg/kg/day based on our food survey. They noted that estimated exposures from all sources were higher for infants than older children and adults, approximately 10 μg/kg/day for DEP and DEHP, whereas we estimated infant dietary intakes of 4.2 μg/kg/day for DEHP and only 0.02 μg/kg/day for DEP. Wormuth et al. (2006) concluded that, for adults, ingestion of food was the dominant exposure pathway for DEHP, DiBP, and DIDP, whereas personal care products dominated for DEP exposure and inhalation pathways dominated for DMP.

In addition to including nondietary sources of exposure, previous estimates may have been higher because of different exposure patterns in other populations or because of differences in the food samples measured to determine average concentrations. Phthalate contaminants in laboratory products may also result in higher estimated concentrations in food samples, as discussed by Guo and Kannan (2012). Phthalates are present in many laboratory products, including plastic materials (e.g., pipette tips), glassware, organic solvents, and sorbents, as well as in laboratory air and dust. Organic solvents used to extract target compounds from foods may also be a major source of phthalates, especially DEHP, DBP, and DIBP. We analyzed several laboratory products for the measurement of phthalate concentrations, as reported in detail elsewhere (Guo and Kannan 2012), and devoted considerable effort to developing a reliable method for measuring phthalates in food that introduces negligible levels of contamination. Despite this, there are several limitations in our analytical method. First, despite our efforts to reduce background levels of contamination, DEHP and DBP were still found in all procedural blanks. However, data were discarded if the concentrations of DEHP and DBP in the three procedural blanks varied widely, and the samples were reanalyzed until reliable and reproducible data were obtained. Second, we applied a conservative approach of subtracting the highest blank values from the concentrations measured in food samples, which may have underestimated the actual concentrations in foods. Third, our analytical method has not been validated for all categories of foods, although it has been validated for oils and fats, which pose the greatest challenges for the analysis of trace levels of lipophilic compounds. In addition, we included labeled internal standards in all food samples analyzed, which should enhance the validity of the analytical results.

While the present study focused only on food exposures, as did our previous studies on other classes of contaminants in food (Schecter et al. 2006, 2010, 2012), the use of phthalates in many consumer products has been recognized within the scientific and regulatory community as an important issue for human exposure. The U.S. EPA’s Office of Pollution Prevention and Toxics’ Existing Chemicals Program (U.S. EPA 2011a) outlines the U.S. EPA’s activities regarding cumulative exposures to phthalates. They identify eight phthalates (DBP, DiBP, BBzP, DnHP, DEHP, DnOP, DINP, and DIDP) for which they are currently obtaining data and determining regulatory options in the context of a Phthalates Action Plan (U.S. EPA 2011c), and all but DnOP and DINP are undergoing an extensive review by the U.S. EPA’s Integrated Risk Information System (IRIS) program that will include assessments of each individual phthalate and a cumulative risk assessment. The National Academies of Science recognized the importance of cumulative exposure—generally and specifically—to phthalates when they released the report, *Phthalates and Cumulative Risk Assessment—The Task Ahead*, in 2008 (National Academies of Science 2008). Finally, the Consumer Product Safety Commission (CPSC) initiated a Chronic Hazard Advisory Panel on Phthalates in 2010 to study cumulative exposure and potential health effects of phthalates, with a particular emphasis on children’s exposure to phthalates in toys (CPSC 2010).

Once these U.S. EPA and CPSC efforts are completed, likely during 2013, more data will be available to estimate cumulative exposures to the phthalates measured in our survey. Until then, there are a limited number of individual benchmarks with which to compare the dietary intakes estimated in this study. Our data suggest that dietary intakes of individual phthalates are much less than currently published U.S. EPA reference dose (RfD) benchmarks, although it is important to note that RfDs pertain to all pathways of exposure. For example, the currently published RfD for DEHP on the U.S. EPA’s IRIS database is 20 μg/kg/day (U.S. EPA 2012c), whereas our estimate of total dietary intake is only 0.7 μg/kg/day. Similarly, our estimated dietary intakes of 0.2 μg/kg/day for DBP, 0.03 μg/kg/day for DEP, and 0.085 μg/kg/day for BBzP are substantially lower than corresponding published RfDs of 100 μg/kg/day (U.S. EPA 2012b), 800 μg/kg/day (U.S. EPA 2012d), and 200 μg/kg/day (U.S. EPA 2012a), respectively. To date, the U.S. EPA has not published RfDs for the other phthalates measured in this study.

This study is a first step in examining U.S. exposures to phthalates from food. We analyzed a limited one-time sample of foods purchased from supermarkets in only one geographic location, and, perhaps more importantly, did not evaluate foods as packaged, processed, or served in homes or restaurants. Future studies will need to focus on the influence of packaging as well as the preparation of foods. Further representative surveys of phthalates in U.S. food are also indicated, as is research on the toxicity of phthalate mixtures in food and from other sources.

## Supplemental Material

(684 KB) PDF
