# Supplementary material for: Phthalate Concentrations and Dietary Exposure from Food Purchased in New York State
Source: Environ Health Perspect. 2013 Mar 6;121(4):473–9. doi: 10.1289/ehp.1206367 (PMC3620091; doi:10.1289/ehp.1206367)
Supplement: (684 KB) PDF [file ehp.1206367.s001.pdf]

Supplemental Material

Phthalate Concentrations and Dietary Exposure from Food Purchased in New York State

Arnold Schecter, Matthew Lorber, Ying Guo, Qian Wu, Se Hun Yun, Kurunthachalam Kannan, Madeline Hommel, Nadia Imran, Linda S. Hynan, Dunlei Cheng, Justin A. Colacino, and Linda S. Birnbaum

Supplemental Material, Table S1. Concentrations of phthalates (ng/g wet wt) measured in foodstuffs from Albany, New York, USA in 2011 for each individual sample. Samples below the level of detection (LOD) are indicated by "*".

Category	Sample	Container Type	DMP (0.2)[a]	DEP (0.2)	DiBP (0.2)	DBP (1.4)	DnHP (0.2)	BBzP (0.2)	DCHP (0.2)	DEHP (3.7)	DnOP (1.0)
Beverages	Diet lemon tea	Plastic	*	*	0.64	*	*	*	*	18.2	*
	Lemonade	Plastic	*	*	*	*	*	*	*	*	*
	Soda	Plastic	0.22	*	*	*	*	*	*	*	*
	Bottled water	Plastic	*	*	*	*	*	*	*	*	*
	Green tea	Plastic	*	*	*	*	*	*	*	*	*
	Sports drink	Plastic	*	*	*	*	*	*	*	*	*
	Sports drink	Plastic	*	*	0.23	*	*	*	*	*	*
	Apple juice	Plastic	0.23	*	0.95	*	*	*	*	*	*
Milk	Milk	Plastic	*	*	*	*	*	*	*	28	2.52
	Milk	Plastic	*	0.24	0.3	2.3	*	0.99	*	69.1	*
Other Dairy	Pudding	Plastic	*	*	0.75	31.7	*	*	*	92.8	*
	Sliced cheese	Plastic	0.85	0.66	4.01	138	*	0.49	1.86	69.7	*
	Ice cream	Paper	*	1.91	0.57	4.77	0.21	1.89	*	341	20.8
	Ice cream	Paper	*	0.86	1.81	*	8.82	18.3	*	10.2	*
	Butter	Paper	0.7	0.55	1.19	252	1.58	*	*	275	*
	Butter	Paper	1.54	2.78	0.79	*	*	5.02	*	200	*
	Shredded cheese	Plastic	0.73	5.27	7.87	513	*	10.8	*	285	*
	Yogurt	Plastic	*	*	*	*	*	*	*	3.71	*
	Yogurt	Plastic	*	*	*	*	*	1.2	*	16.6	*
Fish	Salmon fillet	Foam/ plastic	0.43	1.03	*	*	*	2.43	*	54.8	*
	Tuna	Plastic	*	0.93	3.7	47.5	*	*	*	39.6	*
	Raw shrimp	Plastic	*	*	*	*	*	*	*	*	*
	Chopped clams	Metal	*	*	*	*	*	*	*	6.53	*

Category	Sample	Container Type	DMP (0.2)[a]	DEP (0.2)	DiBP (0.2)	DBP (1.4)	DnHP (0.2)	BBzP (0.2)	DCHP (0.2)	DEHP (3.7)	DnOP (1.0)
	Sardines	Metal	0.32	0.86	0.99	5.5	0.25	5.32	*	55.8	*
Fruits/Vegetables	Tomato sauce	Metal	*	0.2	1.2	*	*	*	*	*	*
	Canned fruits	Plastic	*	*	*	*	*	*	*	*	*
	Vegetable soup	Plastic	*	*	0.48	*	*	0.34	*	21.7	*
	Vegetable soup	Plastic	*	*	0.67	*	*	2.72	*	*	*
	Canned fruits	Metal	*	*	0.28	*	*	*	*	3.74	*
Grain	Cookies	Paper	0.34	0.75	4.01	11.7	*	1.95	*	151	*
	Cake mix	Paper	*	0.45	0.68	5.14	0.27	7.25	*	41.5	*
	Cereals	Paper	*	2.72	14	30.6	*	4.76	*	20	*
	Cookies	Plastic	0.29	1.17	*	*	0.34	0.5	*	39.4	*
	Bread	Plastic	0.44	78.8	1.06	3.35	0.61	2.1	*	69.6	*
	Cookies	Paper	0.35	0.43	3.27	57.9	*	20.2	*	50.6	*
	Rice	Paper	0.47	3.55	1.64	1.7	*	4.65	*	59.1	*
Beef	Ground beef	Plastic, paper	0.25	0.83	*	*	4.83	1.11	*	*	*
	Beef	Foam/ plastic	*	0.45	*	*	*	*	*	*	6.64
Pork	Sausage links	Foam/ plastic	*	0.22	*	*	*	*	*	8.16	*
	Pork	Foam/ plastic	0.89	0.6	*	*	*	*	*	*	*
	Pork bacon	Plastic/ paper	0.21	0.82	24.7	*	*	*	*	33.1	*
	Ham	Plastic	*	0.57	*	*	*	0.6	*	1158	9.95
Poultry	Ground turkey	Foam/ plastic	0.2	0.32	*	*	*	*	*	22.6	*
	Ground chicken	Foam/ plastic	0.21	0.74	*	*	*	*	*	7	*
	Chicken drumsticks	Foam/ plastic	*	0.34	*	*	*	1.99	*	6	*
	Turkey breast	Plastic	*	0.73	*	*	*	*	*	36.4	*
	Turkey bacon	Plastic/ paper	*	0.25	*	*	*	*	*	38	*
	Chicken franks	Plastic	0.21	*	*	*	0.73	1.58	*	*	*
Hotdog	Hotdog	Plastic	0.3	0.39	*	*	*	*	*	6.36	*
Vegetable Oils	Canola oil	Plastic	*	*	*	*	*	2.2	*	*	*

Category	Sample	Container Type	DMP (0.2)[a]	DEP (0.2)	DiBP (0.2)	DBP (1.4)	DnHP (0.2)	BBzP (0.2)	DCHP (0.2)	DEHP (3.7)	DnOP (1.0)
	Virgin olive oil	Glass	3.41	*	9.26	9.2	*	459	42.6	300	*
	Vegetable oil	Plastic	*	*	0.25	*	0.37	0.35	*	48.9	1.51
Condiments	Pudding syrup	Plastic	*	*	0.44	*	*	0.3	*	21.2	*
	Marinade	Glass	0.29	*	1.03	6.66	*	3.34	0.29	7.36	*
	Barbecue sauce	Plastic	*	*	*	*	*	*	*	*	*
	Italian dressing	Plastic	0.86	0.29	2.82	80.9	*	*	*	103	*
	Ranch	Plastic	0.5	3.8	0.66	2.5	*	2.35	*	29.1	4.61
	Tomato ketchup	Plastic	*	0.21	0.95	*	*	5.77	*	20	*
Infant Food	Rice cereal	Paper	*	0.54	2.71	2.93	*	1.22	0.69	95.2	*
	Readymade meal for baby	Plastic	*	0.4	0.22	1.55	*	11.7	*	105	6.13
	Readymade meal for baby	Plastic	*	0.92	0.95	*	*	3.5	*	29.4	*
	Fruit homogenate	Plastic	*	0.28	1.19	*	*	3.53	*	15.8	*
	Vegetable homogenate	Plastic	*	*	0.11	*	*	1.13	*	23.8	*
	Fruit homogenate	Glass	*	*	*	*	*	2.37	*	235	8.85
	Food homogenate	Glass	*	*	*	*	*	*	*	21.2	*
Measured, but not included in other food categories	Chicken noodles	Plastic	*	0.47	1.18	39.6	*	4.7	1.56	25.3	*
	Chicken pot pie	Paper plate, paper box	*	1.37	*	*	*	0.83	*	32.1	*
	Pizza	Plastic	0.47	3.61	*	*	*	0.94	*	63.2	*
	Turkey & dressing with gravy	Plastic	0.22	1.29	2.91	14.7	0.6	30.6	*	175	26.1
	Instant noodles	Plastic	0.51	1.36	2.5	74.7	*	*	*	41.6	*
	Onion soup mix	Paper	0.33	2.68	0.39	1.58	*	1.41	*	138	*
	Noodle soup	Metal	*	*	*	*	*	1.69	*	7.56	*

[a] The limit of detection for each phthalate ester is indicated in parenthesis.
